# Salivary ⍺-Amylase Time-Effect on the Main Groups of Thickening Products Intended to Manage Patients with Oropharyngeal Dysphagia

**DOI:** 10.3390/foods14223829

**Published:** 2025-11-08

**Authors:** Adrian Nuñez-Lara, Alberto Solís, Irene Domínguez-López, Begoña Murga-Jambert, Pere Clave, Mireia Bolivar-Prados

**Affiliations:** 1 Gastrointestinal Physiology Laboratory, Hospital de Mataró, Universitat Autònoma de Barcelona, 08304 Mataró, Spain; anunezla@csdm.cat (A.N.-L.); mbolivar@csdm.cat (M.B.-P.); 2 Centro de Investigación Biomédica en Red de Enfermedades Hepáticas y Digestivas (Ciberehd), 08304 Barcelona, Spain; 3 Gastroenterology Department José de Jesus Villalobos Pérez, Instituto Nacional de Ciencias Médicas y Nutrición Salvador Zubirán, Mexico City 14080, Mexico; beto_chilout@hotmail.com; 4 Escola Santa Maria dels Apòstols, Misioneras Cruzadas de la Iglesia, 08019 Barcelona, Spain; irenedominguezlopezz@gmail.com (I.D.-L.); begomurgajambert@gmail.com (B.M.-J.)

**Keywords:** dysphagia, thickening product, viscosity, rheology, amylase, shear rate

## Abstract

Shear viscosity is the main property linked to the therapeutic effect of thickening products (TP) and can be reduced by the effect of salivary α-amylase during the oral phase and shear thinning during the pharyngeal phase of swallowing. The main aim of this study was to determine the time-effect salivary α-amylase. The study solutions were prepared with three types of TP: modified starch (MS), xanthan gum (XG), and mixture (MX), and two viscosity levels: 250 and 800 mPa·s at 50 s^−1^. Five volunteers performed oral incubations from 5 to 60 s to assess the time-effect of α-amylase. The results revealed that MS-TP presented a sudden reduction in shear viscosity >99% at 5 s at both viscosity levels. In contrast, XG-TP showed only a slight reduction (1–20%) to α-amylase for all the time intervals. MX-TP viscosity exhibited a reduction of 25% for 250 and 13% for 800 mPa·s. The immediate and extreme reduction in shear viscosity of MS-TP in contact with α-amylase contrasted with the amylase resistance presented by TPs that contained XG. These findings improve the description of the full rheological behavior of TP and provide valuable insights into optimizing the choice of TP in the management of patients suffering from swallowing disorders.

## 1. Introduction

Oropharyngeal dysphagia (OD) is a disorder consisting of difficulty forming or moving or the alimentary bolus from the mouth to the esophagus [1], classified under the International Classifications of Diseases (ICD-11) under the code MD93. OD is highly prevalent in older individuals [2] and it is associated with several complications which reduce the life quality [3] such as malnutrition, dehydration, and aspiration pneumonia [4,5].

Current treatment has largely focused on compensatory management, including the increment of viscosity for fluids [6,7] and the adaptation of texture for solids [8]. Fluid viscosity is usually increased with thickening products (TP), which has scientific evidence as a valid and effective strategy to reduce the risk of airway invasion [7,9,10]. Shear viscosity is the main parameter involved in the therapeutic effect of TP [9,10] and measures the resistance of a fluid to flow, expressed in SI units (mPa·s) [11]. However, shear viscosity can be affected by two main factors when swallowing: the salivary α-amylase enzyme in the oral phase [12,13,14,15] and shear rate in the pharyngeal phase [15,16,17]. The effect of these two factors is directly related to the hydrocolloid composition of TPs [18] which can be divided into three main groups as follows: modified starch (MS), xanthan gum (XG), and a mixture of both (MX) [19,20].

In the oral phase of the swallowing process, the enzyme salivary α-amylase is present in the saliva and helps with food digestion by breaking the O-glycoside bonds [12,15]. This effect can reduce the therapeutic effect of the TP by reducing its shear viscosity [19], especially for MS-based TP which present a high number of α-1→4 bonds in linear chains [21,22]. In contrast, XG-based TP establish β-1→4 bonds in a tridimensional structure which protects and isolates the bonds inside and is less affected by salivary α-amylase [16,23]. It has been observed that the viscosity of MS-based TP is reduced dramatically in the oral cavity [19,24]. Nevertheless, the effect of this enzyme over time in TP has not yet been determined.

In patients with oropharyngeal dysphagia, delayed oral transit prolongs contact between saliva and the thickened bolus, allowing salivary α-amylase to degrade TP’s biochemical structure and markedly decrease viscosity, which may negatively affect swallowing safety.

TPs also suffer reduction in shear viscosity in the pharyngeal phase caused by the increment of shear rate associated with pharyngeal bolus velocity [25]. Shear rate is defined as the rate of shear strain to which the fluid is subjected, with subsequent changes in velocity [19]. TPs are non-Newtonian fluids with a shear thinning behavior, thus they present a decrease in viscosity with the increment in the shear rate [11]. In the swallowing process, shear rate ranges from 1 to 1000 s^−1^ [17] but there are two main values which should be considered for dysphagia management: 50 s^−1^ and 300 s^−1^, which correspond to the shear rate in the oral phase and in the mesopharynx [17].

These effects of both oral and pharyngeal swallowing factors reduce the therapeutic effect of TPs. In order to assess the doses which provide greater safety during swallowing, several dose–response studies with different TPs and OD phenotypes have been performed [9,10]. A strong viscosity-dependent therapeutic effect on the safety of swallow was found at a range between 250 and 1000 mPa·s [9] and 250–800 mPa·s [10], depending on the TP. Two viscosities were found to be optimal for patients with OD: 250 mPa·s and 800 mPa·s, which provided safe swallows to more than 90% of the study population [9]. These optimal viscosity levels depend on the impact shear rate and salivary α-amylase have on the bolus, emphasizing the need to study and analyze their effects on TPs. The shear rate effect can be easily assessed with the use of a rotational rheometer, and a common method to test the effect of salivary α-amylase is to perform an oral incubation to mix saliva with the product [26]. However, there are no studies that assess the relationship between the time-effect of α-amylase in contact with the TP.

The main aims of this study are therefore (a) to assess the time-effect (5–60 s) of salivary α-amylase by oral incubation on the main groups of TP in healthy volunteers, (b) to determine the impact of shear rate at 50 (oral phase) and 300 s^−1^ (pharyngeal phase), and (c) to compare the effect of salivary α-amylase on time between each TP.

## 2. Materials and Methods

### 2.1. Experimental Design

**Figure 1:**
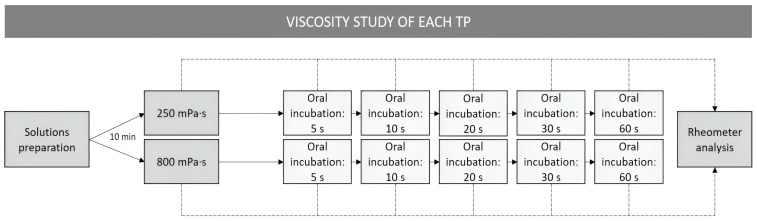
Study design.

This was a reference—controlled, multiple-dose, and single-center, in vitro, and ex vivo observational study. The study design is presented in Figure 1. Three different TPs were selected to perform the study at two viscosity levels (250 mPa·s and 800 mPa·s [9]). Both viscosity levels were analyzed in the rheometer before and after oral incubation in a shear rate range from 0.1 to 1000 s^−1^. Viscosities at 50 and 300 s^−1^ were interpolated to assess viscosity at the oral and pharyngeal phase, respectively [26]. To assess the effect of salivary α-amylase over time, an oral incubation was performed by 5 healthy volunteers at different time intervals: 5 s, 10 s, 20 s, 30 s, and 60 s for both viscosity levels.

### 2.2. Products

Three TP commercialized in Spain were used to perform this study (Table 1): Thicken Up Resource (A; Nestle S. A., Barcelona, Spain), composed of MS; Nutilis Clear (B; Nutricia N. V., Zoetermeer, The Netherlands), composed of XG, maltodextrin, and guar gum; and Fresubin Clear Thickener (C; Fresenius Kabi GmbH, Bad Homburg, Germany), composed of MS, XG, maltodextrin, and modified cellulose. The TPs were divided into three categories according to their main composition (Table 1).

These 3 different TPs have been selected based on previous publication of Bolivar-Prados 2022 [19], in which the rheological patterns of TP were determined. The 10 most commercialized TPs in Spain were selected and 3 main rheological patterns were identified: pattern 1 showed a viscosity of 60–100% in the oral phase (caused by salivary amylase) and between 50 and 70% due to the shear rate effect (50–300 s^−1^); pattern 2 exhibited a reduction of 20–46% and 70–80% and pattern 3 between 0 and 20% and between 70 and 80% at the oral and pharyngeal phase, respectively. Therefore, we selected one TP from each rheological pattern based on their composition: pattern 1 corresponds to MS-based TP, for which we selected Resource Thicken Up. Pattern 3 corresponds to XG-based TP, represented by Nutilis Clear. For pattern 2, which corresponds to a mixture-composition TP, we selected Fresubin Clear. It is important to note that the rheological pattern of mixture-composition TP can vary depending on their composition of thickening agents. In our case, Fresubin Clear was selected because it displayed pattern 2 in the study, representing a typical mixture-based TP.

The preparation process of the solutions was performed according to the protocol published by our group [26]. Briefly, the solvent was weighed in a glass beaker; the TP was weighed and added to the dissolver over 5 s while stirring at 4 rps with a metallic spatula and continued for 30 s at the same velocity. The solution was left to rest for 10 min. Viscosity was analyzed by increasing the shear rate from 0 to 1000 s^−1^ at 25 °C. Viscosity measurements were performed in replicates of five samples for each viscosity level.

The dose (g/100 mL mineral water) of each TP to achieve 250 and 800 mPa·s is represented in Table 2. TP A needed 5.5 and 6.5 g, for each viscosity level, respectively. TP B and C reduced the quantity of thickening product (34–73%) to achieve the same viscosity levels.

### 2.3. Participants

Five healthy volunteers were asked to participate in the study. Accepting an alpha risk of 0.05 and a power of 0.1 in bilateral contrast, it was calculated that 5 participants were necessary to recognize a statistically significant difference greater than or equal to 0.05 units.

Main inclusion criteria were to be between 18 and 40 years, not present mastication or swallowing impairments, and to accept and sign informed consent. Main exclusion criteria were to have a severe cognitive disorder or uncertainty on the part of the investigator about the willingness or ability of the participant to accomplish the protocol requirements and instructions, irritation or inflammation of the oral cavity, being unable to undergo the procedure, and/or allergy to any ingredient of the study products.

Before each oral incubation, participants rinsed their mouths with mineral water for 10 s to remove any residues from previous tests. Then, 15 mL of the TP solution were administered, and participants were instructed to keep the bolus in the oral cavity for the indicated time (5, 10, 20, 30, or 60 s) without chewing or swallowing, while performing slow and gentle tongue movements to allow uniform mixing with saliva. As suppression of the swallowing reflex was not feasible, the administered volume exceeded the amount required for rheological testing to ensure adequate sample recovery. Moreover, previous studies report that the standard bolus volume for healthy adults is approximately 15 mL in women and 18.8 mL in men [27], indicating that our approach is representative of realistic conditions for patients with OD.

At the end of each period, the bolus was expectorated into a sterile container and immediately transferred to the rheometer for viscosity determination. The viscosity measurement was initiated within approximately 30 s after expectoration, to preserve the physiological conditions of salivary α-amylase activity. All samples were handled under ex vivo precautions to ensure hygienic and consistent processing. This procedure was repeated for both viscosity levels.

### 2.4. Equipment

To assess the shear viscosity, a rotational rheometer Anton Paar RheolabQC (Graz, Austria) was used. Two types of rotor sensory system were selected: DG42/SS/QC, designed with a larger sample contact area, making it especially accurate for measuring low-viscosity fluids (<300 mPa·s); CC17/QC, with a more general-purpose design that allows it to accurately analyze high-viscosity fluids (>300 mPa·s), as indicated by the manufacturer [28]. Therefore, DG42/SS/QC was selected for samples with an expected viscosity of 250 mPa·s, and CC17/QC for samples with an expected viscosity of 800 mPa·s. Data were analyzed by RheoCompass (version 1.30. Ink) software. The temperature was set at 25 °C on all tests, in order to prevent the interaction between TP and α-amylase from being altered by external temperature fluctuations. Although the oral cavity temperature is approximately at 37 °C and a higher temperature may slightly increase enzymatic activity, the bolus does not adapt to this temperature during the short oral incubation interval of just 5–60 s. Therefore, the results obtained at 25 °C likely represent a conservative estimation of the in vivo α-amylase effect. Shear viscosity values were expressed in SI units (mPa·s).

### 2.5. Data Analysis

Continuous data are presented as mean ± standard deviation. Quantitative data have been used to describe the decrease in viscosity through salivary amylase or shear rate and is presented in percentage as absolute frequencies.

Effect of salivary α-amylase on viscosity during oral incubation is presented. Decrease in shear viscosity in the oral cavity caused by the effect of salivary α-amylase was calculated with the following formula:

Amylase effect = [(viscosity at 50 s^−1^ − viscosity at 50 s^−1^ after oral incubation)/viscosity at 50 s^−1^] × 100 (1)

Effect of shear thinning on viscosity during pharyngeal flow is presented. Decrease in shear viscosity due to the shear rate was assessed according to the following formula:

Shear thinning effect = [(viscosity at 50 s^−1^ − viscosity at 300 s^−1^)/viscosity at 50 s^−1^] × 100 (2)

Effect on viscosity of both salivary α-amylase and shear thinning during pharyngeal flow is presented. Decrease in viscosity due to the combined effect of the salivary α-amylase and the shear rate was assessed according to the following formula:

Both factors effect = [(viscosity at 50 s^−1^ − viscosity at 300 s^−1^ after oral incubation)/viscosity at 50 s^−1^] × 100 (3)

### 2.6. Statistical Analysis

The time-effect of salivary α-amylase on shear viscosity was analyzed by comparing pre-oral incubation viscosity of each sample with post-oral incubation values obtained at 5, 10, 20, 30, and 60 s. As the same five volunteers were used for all measurements, a repeated-measures one-way ANOVA (RM-ANOVA) was applied to evaluate differences over time.

Given the small sample size (*n* = 5), a non-parametric Friedman test was also performed as a complementary analysis. Both tests showed the same decreasing trend in viscosity, although *p*-values differed slightly due to the reduced power of non-parametric tests. Therefore, the RM-ANOVA results were retained as the main analysis.

Significant differences were considered at *p* < 0.05. All statistical analyses were performed with GraphPad Prism 6.0 (GraphPad Software, San Diego, CA, USA).

## 3. Results

### 3.1. Shear Viscosity

Viscosity values for all TPs with the doses previously determined ranged from 247.40 to 278.80 and 825.38 to 857.66 mPa·s for 250 and 800 viscosity levels, respectively, at 50 s^−1^. No significant differences were observed between TPs for each viscosity level achieved (*p* values between 0.2552 and 0.9841).

### 3.2. Salivary α-Amylase Time-Effect

#### 3.2.1. MS-Based (TP A)

Shear viscosity of TP A after oral incubation ranged between 0.80 ± 0.35 mPa·s and 1.20 ± 0.22 mPa·s for both viscosity levels assessed (Table 3). Viscosity was reduced more than 99% at the very first oral incubation time assessed. A significant (*p* < 0.0001) reduction in shear viscosity was found at all the time intervals of both viscosity levels when compared with pre-oral incubation viscosity values. No significant differences were found between post-oral incubation samples at either 250 or 800 mPa·s (*p* > 0.9999).

#### 3.2.2. XG-Based (TP B)

Viscosity after oral incubation experimented a maximal decrease of 17.22 and 12.72%, for 250 and 800 mPa·s, respectively (Table 4). A slight significant reduction was only observed for 250 mPa·s at 60 s post-oral incubation respecting pre-oral incubation (*p* = 0.0326). No significant differences were found between post-oral incubation samples at either 250 (*p* values between 0.5945 and >0.9999) or 800 mPa·s (*p* values between 0.1421 and >0.9999).

#### 3.2.3. Mixture (TP C)

Shear viscosity was reduced from 171.64 to 204.41 mPa·s and 676.76 to 723.75 mPa·s, at 250 and 800 mPa·s level, respectively (Table 5). Maximal viscosity reduction was 30.62% (250 mPa·s level, 10 s after oral incubation). Significant viscosity reductions were observed for all time-intervals when comparing with pre-oral incubation (*p* values between <0.0001 and 0.0259), except for 10 s post-oral incubation for 800 mPa·s level (*p* = 0.0735). No significant differences were found between post-oral incubation samples at either 250 (*p* values between 0.1325 and >0.9999) or 800 mPa·s (*p* values between 0.8037 and >0.9999).

#### 3.2.4. Comparison Between Thickening Products

**Figure 2:**
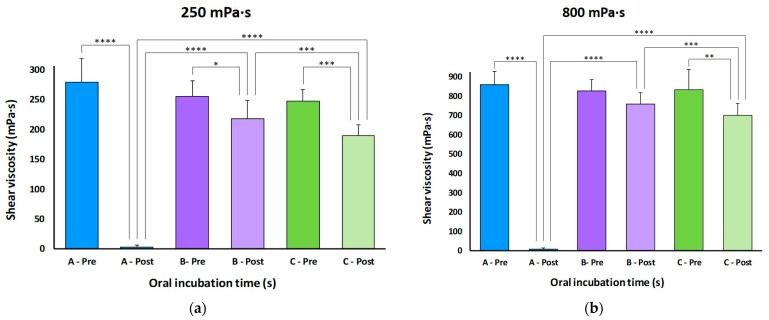
Comparison of the mean viscosity obtained for each thickening product at 250 (**a**) and 800 mPa·s (**b**) level before and after oral incubation. * *p* < 0.05; ** *p* < 0.01; *** *p* < 0.001; **** *p* < 0.0001.

TP A (MS) was extremely affected (>99%) by salivary amylase after 5 s post-oral incubation for both viscosity levels tested (*p* < 0.0001). In contrast, TPs with xanthan gum in their composition (B and C) presented greater viscosity stability when submitted to salivary amylase effect. The viscosity of TP B (XG) at 250 was reduced significantly (*p* = 0.0261) but with a maximal reduction of 19%. No significant reduction was observed at 800 (*p* = 0.0776). The viscosity of TP C (MX) was reduced significantly at both 250 (*p* = 0.0005) and 800 levels (*p* = 0.0023). Comparison of post-oral incubation viscosities for all the TPs assessed showed significant differences (*p* values between 0.0006 and <0.0001). The highest viscosity after salivary amylase reduction was maintained by XG-based TP (B). Figure 2 shows the comparison between all TPs after oral incubation at both viscosity levels.

**Figure 3:**
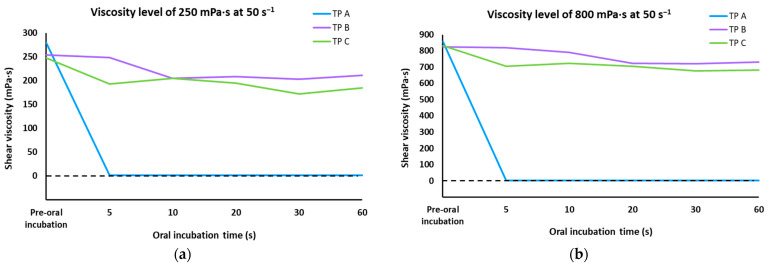
(**a**) Viscosity curves for the three thickening products at a constant shear rate of 50 s^−1^ before and after oral incubation in a time frame of 5 to 60 s for 250 (**a**) and 800 (**b**) mPa·s level.

When assessing shear viscosity over oral incubation time at a constant shear rate, it was observed that TP A sharply decreased at 5 s (to 0–1 mPa·s), while TP B and C maintained stable shear viscosity ranges (±17 and 31%, respectively). Figure 3 shows the viscosity behavior over time after oral incubation at a constant shear rate.

### 3.3. Shear Thinning Effect

Shear thinning effect from 50 to 300 s^−1^ was less pronounced for TP A in comparison to the TPs with XG in their composition (B and C) which is in line with previous studies [19]. Viscosity was reduced by 61 to 64% for TP A and by 73 to 79% for TP B and C.

Viscosity was reduced for all TP at 300 s^−1^ by 60.78 to 108.31 mPa·s for 250 levels and 176.62 to 307.28 mPa·s for 800 levels. Shear thinning was lower for TP A (61 to 64%) compared to TP B and C (76 to 78%). Results are shown in Table 6.

## 4. Discussion

A major concern when using TP is the instability of the viscosity under the salivary amylase effect and the time that the patient needs to prepare the bolus ready to swallow. This study shows distinct patterns of viscosity reduction depending on the type of thickening agent, highlighting the complex interactions between saliva enzymes and the TP composition.

In particular, the interaction between α-amylase and the type of thickening agent is critical. α-amylase specifically hydrolyzes α-1,4 glycosidic bonds in modified starch (MS), rapidly degrading its polymeric structure. In contrast, xanthan gum (XG), a high molecular weight polysaccharide with a β-1,4-glucose backbone and side chains, is resistant to enzymatic breakdown by salivary amylase due to its particular chemical structure [23,29].

The main result arising from this study is the immediate reduction in shear viscosity of MS-based TP (product A) upon exposure to salivary amylase (exceeding 99% reduction in initial viscosity at 50 s^−1^ within the first 5 s of oral incubation). This near-instantaneous viscosity loss is consistent with other critical studies in the field. For instance, Lee et al. (2016) [14] reported a reduction of 99.9% in the initial viscosity of MS-based TP, noting no significant difference in viscosity values between 0 and 60 min of salivary contact, suggesting the degradation is immediate. Similarly, Hanson et al. (2012) [12] observed a 90% viscosity reduction after just 10 s of saliva exposure for a MS-based TP.

When comparing these findings with established therapeutic viscosity levels for patients with OD (250 and 800 mPa·s [9,10]), it is revealed that MS-based TP drops significantly far below safe-swallowing levels almost instantly. For example, product A 800 mPa·s preparation (Table 3) fell to ~1 mPa·s—a viscosity functionally equivalent to water [30]—after only 5 s, eliminating the intended bolus control effect and increasing the risk of oropharyngeal aspiration. Shear viscosity has been determined as the main parameter involved in ensuring safe swallow [6,9,10]; thus, these results raise significant concerns regarding the therapeutic effect of some TPs during the swallowing process. It is true that MS-based TP have also demonstrated their therapeutic effect when being given to patients with OD [1,18]. However, viscosity levels used for this specific TP are usually higher than the ones selected for XG-based TP [18].

The molecular structure of MS, despite being modified to increase its functional stability, remains susceptible to enzymatic hydrolysis due to the presence of digestible α-glucan chains and their mode of action (absorbing water and swelling). In addition, the absence of dietary fiber in product A (Table 1) further supports its lack of structural resistance in the oral cavity, as fiber-rich polysaccharides tend to enhance viscoelastic properties and enzyme resistance [29]. This structural and compositional profile explains the rapid viscosity loss observed in MS-based TP, reflecting the high enzymatic vulnerability of starch-based formulations.

In contrast, the more stable rheological profile of XG is attributed to its resistance to amylase and its ability to form a strong, three-dimensional network in solution. External studies confirm that, while MS-based products are highly susceptible to amylase, XG-based shows little to no responsiveness to enzymatic breakdown [31]. This inherent stability makes it a more reliable vehicle for OD management, as its prescribed shear viscosity is more likely to be maintained during the oral phase [13,14].

According to the percentage of XG included in TP composition, the effect of salivary amylase can vary [19]. In this specific case, the initial viscosity of product B (XG-based TP) was reduced by 12 to 17%, while product C (mixture composition TP) was reduced by 13 to 31%, suggesting that the higher the quantity of XG in the composition of the product, the lower the effect of salivary amylase over viscosity. In addition, the high dietary fiber content of product B (derived from xanthan gum and guar gum, Table 1) and C (derived from xanthan gum and modified cellulose, Table 1) is also likely to contribute to its increased structural integrity and stability against enzymatic breakdown [32].

From a clinical standpoint, these differences are highly relevant. XG-based TP maintained shear viscosities within or near the 250 and 800 mPa·s levels even after 60 s of oral incubation (Table 4), making them suitable for patients with delayed oral transit or impaired tongue propulsion. In contrast, MS-based TP would require higher initial viscosities to remain within the viscosity therapeutic window after 5 s, which could reduce palatability and patient acceptability. The mixture-composition TP (product C) showed intermediate stability; however, for 250 mPa·s preparations, post-incubation values (for example, 172 mPa·s at 30 s) fell near to the lower limit of the shear viscosity range with therapeutic effect on swallowing safety (150–800 mPa·s) [9], potentially increasing aspiration risk in patients with prolonged swallowing.

This finding provides further evidence that partial or total inclusion of XG in the TP formula may increase shear viscosity stability, both in absolute values and over the time in contact with amylase. This could be crucial for patients with OD, by enhancing swallowing safety and reducing the risk of aspiration.

These conclusions are supported by recent clinical literature. A systematic review by Hadde et al. in 2021 on the safety and efficacy of XG-based TP [33] concluded that they improve swallowing safety by reducing aspiration risk, providing greater bolus cohesion and stability, and showing high resistance to α-amylase, without increasing pharyngeal residue.

Another factor to take into account is the time that the patient needs to prepare the bolus for swallowing, which can also affect the TP properties and, therefore, their therapeutic effect. This study examined the effect of the time needed by the patient to prepare the bolus before swallowing it (which could be altered due to efficacy impairments) on the shear viscosity of the TP. Results demonstrated that shear viscosity of MS-based TP (product A) is not significantly influenced by the time that it is in contact with salivary amylase, as it is reduced in the first seconds. This finding is important for clinical practice, as it suggests that patients may not be able to prevent the loss of viscosity by taking less time to prepare the bolus, further emphasizing the need for more stable formulations, such as those based on XG, which showed better resistance to enzymatic breakdown. This aligns with the ESSD white paper on bolus viscosity (2021) [34], which states that increasing viscosity is an effective compensatory strategy to reduce airway invasion, but its benefits depend on maintaining viscosity until the moment of swallowing, especially in patients with delayed initiation.

As predictable, all TPs experienced a reduction in viscosity according to their shear thinning behavior. Product A presented greater shear rate resistance (61–64%) than the TPs with XG (72–78%), which could be due to its mode of action: while MS absorbs water through its granules and swells [18,19,35], XG forms a stable network with water [16]. When stress is applied, the molecules of XG align with the direction of the applied shear stress, reducing the internal resistance to flow and viscosity is therefore reduced. In contrast, MS granules are macroscopic structures with a highly complex internal organization, a semi-crystalline structure [7,16]. This structure confers an internal non-crystalline zone and the outer zone is crystallized [7,35]. In addition, MSs are submitted to different mechanical processes to modify the functional properties of starch and develop more stable products (modified-starch) [7]. Although MS TPs exhibit a lower viscosity reduction due to shear rate, it loses over 99% of its viscosity in the oral cavity when mixed with saliva. Therefore, the slightly higher shear rate resistance of MS-based TP is clinically irrelevant if viscosity is already compromised below functional levels in the oral phase [36]. This is in agreement with previous studies such as that of Vallons et al. 2015 [13], which demonstrated that a gum-based TP retained significantly higher viscosity after 10 and 20 s of oral processing compared to a starch-based TP, confirming the superior resistance of gum formulations to salivary α-amylase degradation.

This study presents some limitations, such as using only mineral water as a dispersion medium to evaluate the relationship between TP and α-amylase. It is important to recognize that other commonly used fluids (such as juice, milk, or carbonated drinks) may influence the behavior of TPs due to the involvement of a major number of interactions. Previous research suggests that XG maintains more stable viscosity across various food matrices than MS [37,38]. Thus, further research is needed to evaluate product behavior in real-life conditions to improve the applicability and realism of this study’s findings. It is also worth noting that other rheological parameters play important roles in the swallowing process. These aspects could influence bolus formation and rheological behavior [30]. Future studies should also explore the impact of more variables, like oscillatory rheology, extensional flow, or tribological parameters, to better characterize the therapeutic suitability of TPs under realistic oral conditions.

It is also important to note that, although this study was conducted under controlled laboratory conditions, the findings are clinically relevant, as they highlight the rapid loss of viscosity of MS-based products upon salivary contact. Future research should validate these results in patients with OD, comparing swallowing safety and efficiency between formulations.

In summary, the rheological properties of TPs are critical in determining their therapeutic effect. The strong and quick reaction of MS to salivary amylase raises important questions about its effectiveness, while the structural resistance of XG to enzymatic and mechanical breakdown makes it more suitable for maintaining safe swallowing conditions. These findings are significant for the design of food products or medical formulations requiring specific viscosity profiles and stability under oral and pharyngeal conditions. Further studies are needed to explore the long-term effects of different thickening agents and their interactions with saliva, with a focus on optimizing formulations for improved therapeutic outcomes in dysphagia management. There is also a need to explore how thickening products behave over time under varying alimentary fluid, oral conditions, and analyzing more rheological or tribological parameters. This will provide valuable insights into optimizing the choice of TP to improve the management of patients suffering from swallowing disorders.

## 5. Conclusions

- MS-based TP undergoes an immediate and extreme reduction in shear viscosity (over 99%) within just 5 s of oral contact, severely compromising their therapeutic efficacy during the swallowing oral phase.
- XG-containing TPs, whether used alone or in combination with MS, show significantly lower viscosity reductions (ranging from 0 to 15% for XG and 15 to 30% for MX), maintaining greater stability over time.
- These differences are explained by the molecular structure of the components: (a) MS contains α-1,4 glycosidic bonds that are easily hydrolyzed by salivary α-amylase; (b) XG forms a tridimensional network with β-linkages, resistant to enzymatic breakdown, and a higher fiber content which contributes to its superior stability.
- These findings suggest that XG-based TP are more suitable for patients with OD, particularly those with prolonged bolus preparation time or impaired swallowing efficacy (oral phase impairment).
- Future research should investigate the behavior of TP in different fluids and conditions, and to include additional rheological parameters. Research focused on TP behavior during swallowing is crucial for improving the treatment of patients with OD, ultimately having a significant impact on their health outcomes.

## Figures and Tables

**Table 1:** Thickening products selected for the study and their qualitative and nutritional composition.

Thickening Product	Code	Qualitative Composition	Nutritional Composition (100 g)	Production Category
Thicken Up Resource	A	Modified starch	Energy: 356 kcal Carbohydrates: 89 g Dietary fiber: 0 g Protein: 0 g Salt: <0.55 g	Modified starch (MS)
Nutilis Clear	B	Maltodextrin, xanthan gum, guar gum	Energy: 290 kcal Carbohydrates: 57.6 g Dietary fiber: 28 g Protein: 0.8 g Salt: 3.8 g	Xanthan gum-based (XG)
Fresubin Clear	C	Modified starch, xanthan gum, maltodextrin, modified cellulose	Energy: 264 k kcal Carbohydrates: 41.5 g Dietary fiber: 48 g Protein: 0.6 g Salt: 3.8 g	Mixture (MX)

**Table 2:** Doses and solvent to achieve 250 mPa·s and 800 mPa·s viscosity levels for each thickening product.

	Grams of Thickening Product/100mL Mineral Water		
Viscosity Level (mPa·s)	A	B	C
250	5.5	2.4	1.5
800	6.5	4.3	2.7

**Table 3:** Shear viscosity values at 50 s^−1^ after oral incubation and amylase effect at each oral incubation time for thickening product A at 250 and 800 mPa·s levels.

Thickening Product	Viscosity Level	Time	Shear Viscosity at 50 s^−1^ (mPa·s)	Amylase Effect (% Reduction)
A (*n* = 5)	250	Pre-oral	278.80 ± 39.97	-
		5 s	1.11 ± 0.22	99.60
		10 s	1.12 ± 0.50	99.60
		20 s	0.93 ± 0.13	99.67
		30 s	0.87 ± 0.13	99.69
		60 s	0.83 ± 0.14	99.70
	800	Pre-oral	857.66 ± 69.35	-
		5 s	1.20 ± 0.22	99.86
		10 s	0.80 ± 0.35	99.91
		20 s	1.02 ± 0.73	99.88
		30 s	0.96 ± 0.40	99.89
		60 s	0.96 ± 0.20	99.89

**Table 4:** Shear viscosity values at 50 s^−1^ after oral incubation and amylase effect at each oral incubation time for thickening product B at 250 and 800 mPa·s levels.

Thickening Product	Viscosity Level	Time	Shear Viscosity at 50 s^−1^ (mPa·s)	Amylase Effect (% Reduction)
B (*n* = 5)	250	Pre-oral	254.94 ± 18.19	-
		5 s	232.67 ± 12.19	8.74
		10 s	215.39 ± 24.60	15.51
		20 s	213.80 ± 16.49	16.13
		30 s	216.94 ± 34.01	14.90
		60 s	211.04 ± 12.78	17.22
	800	Pre-oral	825.38 ± 60.01	-
		5 s	819.56 ± 43.97	0.71
		10 s	791.18 ± 38.01	4.14
		20 s	724.70 ± 50.46	12.20
		30 s	720.36 ± 83.63	12.72
		60 s	731.66 ± 75.63	11.35

**Table 5:** Shear viscosity values at 50 s^−1^ after oral incubation and amylase effect at each oral incubation time for thickening product C at 250 and 800 mPa·s levels.

Thickening Product	Viscosity Level	Time	Shear Viscosity at 50 s^−1^ (mPa·s)	Amylase Effect (% Reduction)
C (*n* = 5)	250	Pre-oral	247.40 ± 19.50	-
		5 s	193.14 ± 7.67	21.93
		10 s	204.41 ± 21.92	17.38
		20 s	194.82 ± 24.62	21.25
		30 s	171.64 ± 10.13	30.62
		60 s	184.96 ± 27.03	25.24
	800	Pre-oral	832.38 ± 65.02	-
		5 s	705.66 ± 44.88	15.22
		10 s	723.75 ± 52.58	13.05
		20 s	705.83 ± 63.79	15.20
		30 s	676.76 ± 30.66	18.70
		60 s	683.04 ± 83.02	17.94

**Table 6:** Shear viscosity at 300 s^−1^ before and after oral incubation and shear thinning effect for all thickening products at 250 and 800 mPa·s viscosity levels.

Thickening Product	Viscosity Level	Shear Viscosity Pre-Oral at 300 s^−1^ (mPa·s)	Shear Viscosity Post-Oral at 300 s^−1^ (mPa·s)	Shear Thinning (% Reduction)
A (*n* = 5)	250	108.31 ± 12.31	1.20 ± 0.15	61.15
	800	307.28 ± 17.79	1.14 ± 0.24	64.17
B (*n* = 5)	250	60.78 ± 4.70	54.86 ± 5.92	76.16
	800	176.62 ± 14.03	162.14 ± 14.60	78.60
C (*n* = 5)	250	66.06 ± 5.87	50.50 ± 6.49	73.30
	800	187.10 ± 13.50	154.66 ± 14.77	77.52

## Data Availability

The original contributions presented in the study are included in the article. Further inquiries can be directed to the corresponding author.

## Abbreviations

The following abbreviations are used in this manuscript:

OD	Oropharyngeal dysphagia
TP	Thickening products
SI	International System of Units
mPa·s	Millipascal second
MS	Modified starch
XG	Xanthan gum
MX	Mixture of modified starch and xanthan gum
